# Turkish DIRA patient with novel IL1RN gene mutation

**DOI:** 10.1186/1546-0096-13-S1-P181

**Published:** 2015-09-28

**Authors:** A Berdeli, B Sözeri, B Gerceker Türk, A Oz, S Mir

**Affiliations:** 1 Ege University Medical Faculty, Pediatric Department and Moleculer Medicine, Izmir, Turkey

## Introduction

Deficiency of the interleukin-1 receptor antagonist (DIRA)(OMIM 612852) is a recently described rare autoinflammatory and autosomal recessive disease, caused by loss of function mutations in interleukin-1-receptor antagonist gene (IL1RN) leading to the unopposed activation of the IL-1 pathway. The human IL1RN gene is localized to the long arm of chromosome 2 at band 2q13 (OMIM 147679). Until now, only thirteen cases resembling this have beeen reported in the world.

In this study, we described clinical and molecular date male child who had clinical signs of DIRA syndrome firstly analysed in Turkey.

## Materials and methods

Genomic DNA was obtained from peripheral blood. All exon and intron of IL1RN gene were analysed by PCR and direct DNA sequencing method. Furthermore the obtained nucleotid sequence compaired with reference sequence published in NCBI (NM_173841.2).

## Results

The patient is a boy child, was born in 2007 and he is 7 years old now. Since birth he has had skin lesions like erythema and pustules, and was put different clinical diagnosis in different dermotology clinics. He was admitted to our center with the same recurrent skin lesions, erythema covered his entire body, scaling and crusting manifestations in 2013.

Molecular analysis of IL1RN gene revealed a single homozygous C nucleotide deletion at nucleotide position 396 (c.396delC). The novel c.396delC mutation that was found in our study caused frameshift mutation and as a result, stop codon of IL1RN at c.534* position disappeared and the respective protein became non-functional.

## Conclusion

In our laboratory, it is the first case that the accurate genetic diagnosis of a case considered as DIRA has been confirmed with whole sequence analysis of IL1RN gene. Also the patient has been provided with appropriate biological targeted therapy with Anakinra, that is specific molecule for IL1B inhibition.

